# Allergen immunotherapy on the way to product-based evaluation—a WAO statement

**DOI:** 10.1186/s40413-015-0078-8

**Published:** 2015-09-16

**Authors:** Claus Bachert, Mark Larché, Sergio Bonini, Giorgio Walter Canonica, Thomas Kündig, Desiree Larenas-Linnemann, Dennis Ledford, Hugo Neffen, Ruby Pawankar, Giovanni Passalacqua

**Affiliations:** Upper Airway Research Laboratory, University of Ghent, Ghent, Belgium; Firestone Institute for Respiratory Health, Department of Medicine, St. Joseph’s Hospital Healthcare, McMaster University, Hamilton, Canada; Second University of Naples, Rome, Italy; Allergy and Respiratory Diseases, DIMI Department of Internal Medicine, University of Genova, IRCCS AOU S. Martino, Genova, Italy; Department of Dermatology, University Hospital Zurich, Zürich, Switzerland; Hospital Medica Sur, Toriello Guerra, Delegacion Tlalpan, Mexico, DF Mexico; Morsani College of Medicine, University of South Florida, Tampa, Florida USA; Head, Respiratory Medicine Unit, Orlando Alassia Hospital, Santa Fe, Argentina; Division of Allergy, Department of Pediatrics, Nippon Medical School, Tokyo, Japan

## Abstract

Allergen immunotherapy (AIT) is widely used in clinical practice for patients with moderate to severe allergic rhinitis due to inhalant allergens and may be delivered via subcutaneous (SCIT) and sublingual routes (SLIT). However, the quality of evidence for individual AIT products is very heterogeneous, and extensions of overall conclusions (“class effects”) on the efficacy and disease-modifying effects to all AIT products are unjustified. In contrast, each product needs to be evaluated individually, based on available study results, to justify efficacy and specific claims on sustained and disease modifying effects per allergen and targeted patient group (children vs. adults, allergic rhinitis vs. asthma). WAO intends to support the current development to evidence-based AIT, which ultimately will lead to a more efficacious treatment of allergic patients and the appropriate recognition of AIT.

Allergen immunotherapy (AIT) is a recommended therapeutic option for patients with moderate to severe allergic rhinitis with, or without, mild to moderate asthma due to inhalant allergens [1–4]. AIT is considered a disease-modifying treatment based on the long-term symptom relief, even after cessation of treatment. Furthermore, AIT has a documented ability to prevent both the onset of new allergic sensitizations and the development of comorbidities of allergic rhinitis like allergic asthma [5].

There are currently two routes of administration of AIT: subcutaneous (SCIT) and sublingual immunotherapy (SLIT). Several clinical trials and metaanalyses provide evidence to support the efficacy and safety of AIT and its widespread use in clinical practice [6]. However, the many trials are very heterogeneous in nature, especially in terms of the type and quality of allergen product used, treatment schedules and the target populations. Consequently, there is a considerable degree of heterogeneity of results in the metaanalyses [7]. This prevents extension of the overall conclusions (class effect) made with respect of each of the individual products contributing to the metaanalysis. More reliable evidence derived from large clinical trials with a specific product and a commonly used dose schedule is crucial. It is therefore a matter of considerable concern that the efficacy of certain products in clinical trials e.g. demonstrating long-term or disease modifying effects is often claimed as a blanket approval for all products administered via the same application route. In fact, some products have even been marketed without ever being tested by appropriate studies (dependent on the local regulations and the year of introduction), as those studies were not asked for, or ever registered (“named patient products”). Furthermore, some of the products that have been marketed for decades have failed to demonstrate clinical efficacy when tested using standard clinical trials [8].

It is likely that there has been an underreporting of negative results in the past, since only a few clearly negative studies have been published. This highlights the fact that: 1) not all AIT products are effective, or equally effective, and 2) the efficacy claims based on a “class effect”, without supporting evidence for an individual product (involving the design and conduct of rigorous clinical trials), are inappropriate. In fact, it should be understood that the optimistic assumptions for AIT (“class effect”) mentioned above, may be unjustified in a general sense, as the evidence (specifically for long-term efficacy, prevention of asthma and efficacy in children) relies on the results generated with a limited number of products that have undergone well-designed studies. It has been clearly demonstrated in the past that such assumptions may be incorrect with respect to some products that are not well studied [8]. So far, despite the absence of persuasive data from well designed and performed studies, potentially ineffective products continue to be widely used by the medical community.

In the last decade, substantial progress has been made in the development of selected AIT products and based on the current state-of-the-art, there are high quality, adequately powered, double-blind, placebo-controlled, randomized (DBPCR) clinical trials in which a small number of products have been evaluated. From these studies, it is possible to estimate the therapeutic efficacy of specific products as well as their safety profile. On the basis of these studies, recommendations for the use of individual products can be made in specific patient groups. This systematic and rigorous evaluation is mandatory and vital, not only for newly developed products, but also for those already in the market for a long time. For many products, specifically for mixtures of allergens, conclusive and reliable studies have never been performed and claims for their efficacy are unjustified. Furthermore, evaluation needs to be performed not only on a product line basis, but also per allergen, as single products of a product line contain different allergens [8]. Finally, safety and efficacy in children and adults should be evaluated through separate studies.

As an example, an evidence-based evaluation approach was officially adopted in Europe by the German authorities (Federal Ministry of Health and Paul-Ehrlich-Institute, PEI) in 2008, to be followed for all products in the market as named patient products and for products to be newly registered for the treatment of allergies due to the most frequent allergens: grass pollen, tree pollens (birch, hazel, elder), house dust mite (*Dermatophagoides pteronyssinus* and *farinae*), bee and wasp venom (Therapy Allergen Ordinance, Therapie-Allergene Verordnung; TAV 2008). These rules also apply to mixtures involving at least one of the mentioned common allergens. This approach led to the elimination of more than 6,000 products for which efficacy had never been demonstrated. Importantly, these rules do not apply for less frequent allergens (such as rare inhalant or occupational allergens) since the authorities recognize that the financial burden would be too great or study participants are impossible to be found for products with small market indications. Apart from efficacy and safety, the quality of allergen extracts is subjected to control by the authority, including testing every new batch of allergen extract (batch release) for consistency.

Such regulatory prescriptions are intended to ensure the safety and efficacy of frequently used allergen products (at the dose actually recommended by the manufacturer) and aim to exclude ineffective, unsafe, or low quality products from market access. In the medium- to long-term period, these measures will support and encourage the use of AIT, since clear evidence of efficacy and safety is the mandatory basis for the application of AIT in the treatment of allergic patients and for reimbursement. In many countries such regulations do not exist [9], or the approach to AIT differs [10] and, thus, hundreds of AIT products, for which high quality evidence of safety and efficacy have not been collected, continue to be available. Moreover, the terms “SCIT” and “SLIT” are frequently used as “umbrella” descriptions in order to exploit the positive connotations associated with AIT based on positive clinical trials of a limited number of products. Such marketing strategies wrongly imply that products are effective in reducing symptoms and modifying disease, for example, reducing the risk of developing asthma, when there is no objective supportive evidence. This situation is detrimental, as it does not allow physicians and health professionals to base their selection of treatment on individual products which have demonstrated safety and efficacy. At this time, the selection of safe and efficacious products is possible for both SCIT and SLIT, since selected products have been shown to achieve objective evidence of efficacy and safety. It is, therefore, obvious that such products should be preferred whenever possible.

WAO intends to support and encourage this important evidence-based development in the field of AIT for frequently encountered allergens, by providing this document that details the expectations that a product should fulfill, in order to be recommended for safe and effective use. In contrast, differentiation and/or marketing of products merely based on application routes and claims of safety and efficacy for a particular product that has not been substantiated with objective evidence should be discouraged.

The minimum requirements for a product that can be recommended for use, should include publication in the peer-reviewed literature of at least one successful state-of-the-art DBPCR trial in adults for the first year of treatment [11–13], possibly preceded by a dose response/dose finding study (for which provocation testing may be used) to determine the optimal dosage. Such studies should be performed with standardized extracts, with clearly defined doses, being included in clinical trials registers (e.g. clinicaltrials.gov, clinicaltrialsregister.eu), having an adequate sample size/statistical power to detect a meaningful clinical effect (combined symptom and medication scores), following recommendations on appropriate outcome measures, study conduct and reporting as defined in the recent literature [14, 15]. When possible, adherence/compliance should be measured and reported. Allergen products have the capacity to modify the immune system and are considered medicinal products with the need of a marketing authorization. The European Medicines Agency (EMA) is responsible for EU-wide registration of allergen products [16, 17]. The quality, safety and clinical efficacy of allergen products for the authorization processes have to be documented through a straightforward development plan as outlined in the EMA-guidance on the “Clinical Development of Products for Specific Immunotherapy for The Treatment of Allergic Diseases” [18]. Different procedures are followed in other countries such as the US [10]. A robust recommendation for the use of a specific product can be based on a marketing authorization fulfilling the requirements of the Directive 2001 within the EU [19], based on the state-of-the-art proof of the quality, safety and efficacy of the product. These products answer to the requirements in respect of EMA guidelines and undergo a regular pharmacovigilance.

Claims on sustained effects of a product should be based on at least one DBPCR study, with appropriate sample size calculation, over 3 years of treatment. Demonstration of a disease modifying effect is based on studies with a blinded follow-up for at least two consecutive years after AIT discontinuation, while monitoring of symptoms is maintained. Claims for efficacy and safety in children should be based on appropriate DBPCR studies in the pediatric age group. Furthermore, for specific claims, e.g. in asthmatics, appropriate DBPCR studies should be performed in an appropriate patient group, with objective measurements.

In this document, we have refrained from mentioning individual products. Rather, we have attempted to elaborate upon the criteria for evidence-based recommendation of products. This information/evidence can then be used by: [1] physicians as a useful guide to which products to administer to patients for a safe and efficacious outcome and [2] health authorities with regard to reimbursement based on proven cost-effectiveness. As the availability of such products may vary from country to country, an individualized approach may be needed. However, the aim of identifying safe and efficacious products that can be recommended for treatment and reimbursement in individual countries, through an evidence-based evaluation of each individual product, should not be compromised (Tables 1 and 2).

**Table 1:** Reasons for the use of products supported by evidence-based evaluation of safety and efficacy

The efficacy of the product is known and sufficient (it may fulfill the WAO criteria of 20 % over placebo for rhinitis [3] and appropriate criteria for asthma and other organ manifestations)
The safety of the product is known and favorable; risks for the patient can be evaluated
If efficacy and safety in children are known, the usefulness of the product in children can be evaluated
If information on long-term effects is available for the product, theinformation can be used for calculations of the socio-economic impact
If the tolerability or the efficacy in asthma patients is known, the usefulness and risks of the product for therapy in asthmatic populations can be estimated

**Table 2:** Criteria for a recommendable product for SIT

Minimum expectations for a SIT product to be used in adults:
At least one successful state-of-the-art DBPCR trial in adults for the first year of treatment, best preceded by a dose–response study (nasal provocation testing or allergen exposure chambers may be used for the dose finding)
Additional claims can be justified as follows:
Claims on sustained effects of a product should be based on a successful DBPCR study, based on appropriate sample size calculation, over 3 years of treatment
Claims on disease modifying effects: such studies need be followed up blindly for at least two consecutive years without treatment while maintaining monitoring symptoms
Claims for efficacy in asthmatics should be based on an appropriate successful DBPCR study in the appropriate patient group. For claims on tolerability in asthmatics only, the study can also be performed in allergic rhinitis subjects with comorbid asthma.
Minimum expectations for a SIT product to be used in children:
At least one state-of-the-art DBPCR confirmatory trial in children for the first year of treatment
Additional claims can be justified as follows:
Claims on sustained effects of a product should be based on a successful DBPCR study, based on appropriate sample size calculation, over 3 years of treatment
Claims on disease modifying effects: such studies have to be followed up at least two consecutive years without treatment while maintaining monitoring symptoms
